# Heparan sulfate proteoglycans regulate BMP signalling during neural crest induction

**DOI:** 10.1016/j.ydbio.2019.12.015

**Published:** 2020-04-15

**Authors:** James Pegge, Arnold Junior Tatsinkam, Christopher C. Rider, Esther Bell

**Affiliations:** a Centre for Developmental Neurobiology, King’s College London, Guy’s Campus, London, SE1 1UL, UK; b Centre for Biomedical Sciences, Royal Holloway University of London, Egham Hill, Egham, Surrey, TW20 0EX, UK

## Abstract

Bone morphogenetic protein (BMP) signalling is key to many developmental processes, including early regionalisation of the ectoderm. The neural crest is induced here by a combination of BMP and Wnt signals from nearby tissues with many secreted factors contributing to its initial specification at the neural plate border. Gremlin 1 (Grem1) is a secreted BMP antagonist expressed in the neural crest in *Xenopus laevis* but its function here is unknown. As well as binding BMPs, Grem1 has been shown to interact with heparan sulfate proteoglycans (HSPGs), a family of cell surface macromolecules that regulate a diverse array of signalling molecules by affecting their availability and mode of action. This study describes the impact of HSPGs on the function of Grem1 in neural crest induction. It shows for the first time that Grem1 is required for neural crest development in a two-step process comprising an early HSPG-independent, followed by a late HSPG-dependent phase.

## Highlights

• Gremlin 1 is required for neural crest and neural plate border specification.
• HSPGs regulate Gremlin 1 activity *in vivo*.
• Neural crest induction has HSPG independent and dependent phases.

## 1 Introduction

The neural crest is a crucial progenitor cell population arising at the border of the neural plate during vertebrate embryogenesis. Neural crest cells migrate throughout the body and differentiate into diverse derivatives, including melanocytes, craniofacial skeleton, and much of the peripheral nervous system (Simoes-Costa and Bronner, 2015). The neural crest is specified by a combination of signals including BMPs (Barth et al., 1999; Marchant et al., 1998; Nguyen et al., 1998; Tribulo et al., 2003), Wnts (Bang et al., 1999; Chang and Hemmati-Brivanlou, 1998; Garcia-Castro et al., 2002; LaBonne and Bronner-Fraser, 1998; Saint-Jeannet et al., 1997), and FGFs (Monsoro-Burq et al., 2003; Nichane et al., 2008) from the non-neural ectoderm and underlying mesoderm. These signals are integrated at the neural plate border by a set of key transcription factors. For instance, Msx1 responds to both active Wnt signalling and levels of BMP signalling intermediate to those in the neural plate and non-neural ectoderm to promote Pax3 and Zic1 (Monsoro-Burq et al., 2005; Tribulo et al., 2003). Pax3 also responds directly to Wnt signalling (Monsoro-Burq et al., 2005). The combined activity of Pax3 and Zic1 activates specifier genes like *foxd3* to initiate neural crest cell migration and their later differentiation into a wide array of derivatives (de Croze et al., 2011; Milet et al., 2013; Monsoro-Burq et al., 2005; Sato et al., 2005).

Grem1 is a member of the Dan family of BMP antagonists, secreted proteins that directly bind BMPs to obstruct signal transduction (Brazil et al., 2015). Originally identified in *Xenopus*, it was found to be expressed in neural crest cells, although its role in their development remains unknown (Hsu et al., 1998). Like many signalling molecules (Rider and Mulloy, 2017), Grem1 can also bind to heparan sulfate proteoglycans (HSPGs), cell surface macromolecules that may regulate its distribution and biological activity (Tatsinkam et al., 2015, 2017). HSPGs have been shown to mediate Grem1 Vegfr2 agonism, independently from its role in BMP signalling (Chiodelli et al., 2011). The present study details a novel requirement for Grem1 in neural crest induction in *Xenopus* that depends on its ability to bind HSPGs.

## 2 Materials and methods

### 2.1 *In vitro* transcription and morpholino preparation

Plasmids were linearised by restriction digest and RNA for embryo microinjection transcribed using the mMessage mMachine Kit (Invitrogen). Riboprobes for *in situ* hybridisation were transcribed using the DIG RNA Labelling Kit (Roche). Morpholinos (Gene Tools) were prepared by dissolving in autoclaved, double deionised H_2_O to the required concentration for microinjection. Sequence for *grem1* translation-blocking morpholino 1: GCA TAA ACG AGA CAG TTC ATC CTG T. Sequence for morpholino 2: CTG GCT CAC AGC AGA TCA AGA CTA G.

### 2.2 Microinjection

Micropipettes were prepared from glass capillary tubes using a Sutter P-97 needle puller and embryos injected using a Harvard Apparatus PLI-100 microinjector. Injection volume was calibrated by adjusting pulse duration and measuring the size of the droplet in mineral oil over a 0.05 mm graticule. Embryos were immersed in a 3.5% w/v solution of Ficoll (Sigma) in 0.5x Marc’s Modified Ringer’s solution (MMR) for injection to prevent leakage and remained in this solution for at least 4 hours post-injection to allow puncture closure before transfer into 0.1x MMR. For overexpression and knockdown experiments, injection into the animal pole of one hemisphere at the two-cell stage was used to target the ectoderm unilaterally, leaving the contralateral side unaffected, providing an internal control.

### 2.3 Fixation and screening

Embryos were fixed in MEMFA for 10 minutes then washed into PBS for screening and, if necessary, X-gal staining. They were then screened by epifluorescence microscopy. Embryos were selected based on the presence of fluorescent lineage tracer, indicating successful injection uptake, then sorted by location of fluorescence on either the right- or left-hand side, indicating unilateral targeting. Embryos also injected with lacZ were assessed for the presence of β-galactosidase by incubation at 37C with X-gal solution until suitable staining was observed. All embryos were fully fixed for a further 50 minutes in MEMFA to total 1-hour overall fixation. They were then dehydrated by washing twice in methanol and stored at -20C.

### 2.4 *In situ* hybridisation

Embryos were gradually rehydrated by a decreasing methanol series before bleaching. This was followed by six washes in PBS. Embryos were equilibrated in hybridisation buffer at 60C for 1 hour then were hybridised overnight with 1 μg/ml riboprobe.

Excess riboprobe was washed out with fresh hybridisation buffer. Embryos were then washed once in 2x SSC, twice in 0.2x SSC, and once in maleic acid buffer (MAB), prior to blocking in 2% w/v blocking reagent (Roche) in MAB for 1 hour. Embryos were then incubated with anti-DIG antibody conjugated to alkaline phosphatase (Roche) in blocking solution at 4C overnight.

Excess antibody was removed by a series of five MAB washes of 1 hour at room temperature, followed by an additional overnight wash. Embryos were equilibrated in NTMT and colour reaction performed in the dark in NBT/BCIP (Roche) diluted 1:200 in NTMT until suitable staining was observed.

### 2.5 Sectioning

Whole-mount stained embryos for sectioning were fixed in 4% w/v paraformaldehyde at 4C overnight then washed thoroughly in PBS. They were then equilibrated in molten 20% w/v gelatine at 50C. Embryos were embedded in gelatine and the blocks trimmed before fixation in 4% w/v paraformaldehyde for a further 3-5 days then washed in PBS. Blocks were sectioned on a Leica VT1000 S vibratome to a thickness of 50 μm and mounted on slides in 90% v/v glycerol under a coverslip.

### 2.6 qPCR

Total RNA was extracted from whole embryos with 500 μl TRIzol (Invitrogen) and resuspended in autoclaved, double deionised H_2_O. Random hexamer primers were annealed at 65C for 4 minutes then left on ice before cDNA was reverse transcribed with SuperScript II (Invitrogen) at 42C for 30 minutes. qPCR was performed using a SensiMix SYBR Hi-ROX Kit (Bioline) and the following primers: grem1f TGG GCA ATG CAA CTC CTT CT, grem1r GTG GGA GGT TGT AGC TCT GG, odc1f TGC TTT GCT GGT TCT AGT TAC TG, odc1r GTA CCC ATC GAG GCC ACA AA. Amplification was run on a Lightcycler 480 (Roche) in 96-well plates with the following parameters: initial denature 10 minutes 95C, 40 amplification cycles, denature 15 seconds 95C, anneal 30 seconds 60C, elongate 30 seconds 72C, final melt curve to check primer specificity. Expression of *grem1* was normalized to *odc1* and given relative to that at NF stage 36.

## 3 Results and discussion

### 3.1 Endogenous Grem1 expression

To determine the function of Grem1 in neural crest development, we first investigated its endogenous expression. Transcriptomic studies, including microarray (Yanai et al., 2011) and RNA-Seq (Session et al., 2016) approaches have detected early *grem1* expression in *Xenopus* around the time of gastrulation, when neural crest induction occurs. This was verified here using qPCR, which showed the same profile of early expression, followed by a slight fall then a gradual rise (Fig. S1).

**Fig. 1:**
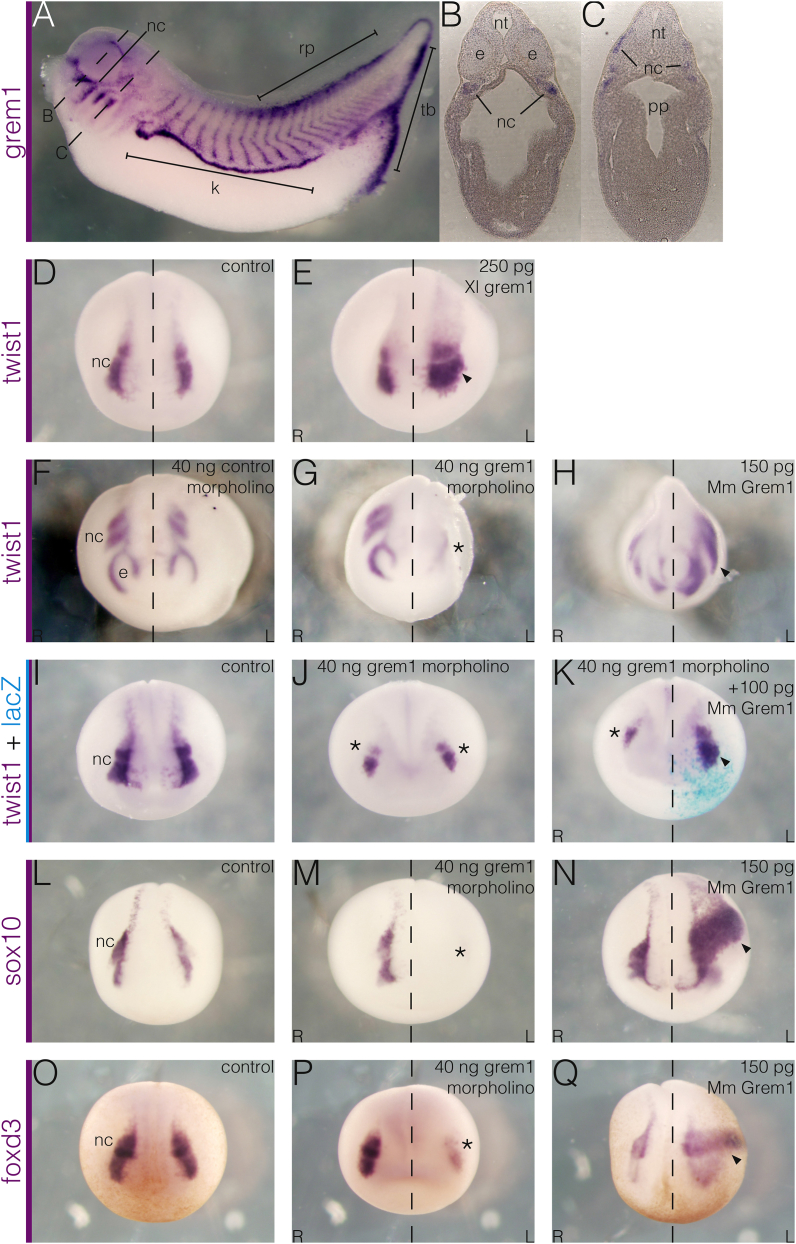
**Endogenous *grem1* expression pattern, and effects of knockdown and overexpression on the neural crest.** (A–C) Stage 36 *grem1* expression pattern. (A) Whole-mount stained embryo showing strong expression in the kidney primordia (k), posterior roofplate (rp) and tailbud (tb), and neural crest (nc). (B-C) Transverse sections from an embryo like the one shown, with approximate anteroposterior locations marked by dashed lines in A. (B) In the most anterior region, *grem1* is expressed in neural crest cells ventral to the developing eye fields (e). (C) In the pharyngeal region, expression is seen in neural crest cells flanking the neural tube (nt) and invading the tissue around the pharyngeal pouches (pp). (D–E) Effect of *grem1* overexpression on the neural crest. (D) Cranial neural crest (nc) around stage 18 is marked by the expression of *twist1*, flanking the anterior neural plate. (E) Overexpression by injection of *Xenopus grem1* RNA causes a substantial expansion (arrowhead) of the neural crest (n ​= ​12/19). (F–G) Effect of *grem1* knockdown. (F) At stage 21 *twist1* marks the neural crest as it begins to surround the eye fields (e) and to migrate in two more posterior streams. It is unaffected by unilateral injection of a standard control morpholino (n ​= ​30/30). (G) Knockdown of *grem1* by injection of a translation-blocking morpholino severely impairs neural crest formation (asterisk, n ​= ​80/93). (H) Injection of murine *Grem1* RNA is also able to expand the domain of *twist1* expression (arrowhead), indicating functional conservation between the mouse and *Xenopus* orthologues (n ​= ​59/74). (I–K) Rescue of neural crest formation with murine *Grem1*. (J) Bilateral *grem1* knockdown depletes the neural crest from both sides (asterisks). (K) Unilateral rescue using murine *Grem1* RNA, mismatched with the morpholino, which only targets the *Xenopus* orthologue, is able to restore neural crest development (arrowhead, n ​= ​14/20). (L–Q) Effect of gain and loss of *grem1* on other neural crest markers. (L) Control neural crest expresses *sox10* (n ​= ​25/25). (M) Unilateral *grem1* knockdown abolishes expression on the injected side (asterisk, n ​= ​19/24). (N) Unilateral overexpression expands the domain of *sox10* (arrowhead, n ​= ​21/30). (O) Control neural crest also expresses *foxd3*. (P) Knockdown similarly abolishes expression (asterisk, n ​= ​24/24). (Q) Likewise, the domain is expanded by overexpression (arrowhead, n ​= ​12/15). Embryo in A is shown in side view with anterior towards the left, sections in B-C are transverse with dorsal towards the top, and embryos in D-Q are shown in frontal view.

Later in development tissue-specific expression is detectable by *in situ* hybridisation (Hsu et al., 1998). At tailbud stages expression is seen in the cranial neural crest (Fig. 1A), within the periocular mesenchyme (Fig. 1B) and dorsal pharyngeal region (Fig. 1C). The function of neural-crest-derived Grem1 is unclear but in chickens it has been linked with the maintenance of forebrain development (Creuzet, 2009), and craniofacial myogenesis (Tzahor et al., 2003). Due to the early onset of *grem1* expression (Session et al., 2016; Yanai et al., 2011), preceding neural fold stages, and its later expression in migratory neural crest cells, we investigated its requirement in initial neural crest specification.

### 3.2 Grem1 is required for neural crest development

To examine the role of Grem1 in neural crest specification we first analysed the expression of *twist1*, a cranial neural crest marker (Hopwood et al., 1989; Fig. 1D). Unilateral overexpression by injection of *Xenopus grem1* RNA caused an expansion in *twist1* expression (Fig. 1E), indicating an increase in neural crest cells. Knockdown was achieved with the use of an antisense, translation-blocking morpholino. While injection of a standard control morpholino had no effect on *twist1* (Fig. 1F), injection of the *grem1* morpholino caused a severe reduction (Fig. 1G), indicating disrupted neural crest formation. This was replicated with a second morpholino to ensure specificity (n ​= ​23/36; data not shown).

To test the functional conservation of Grem1 with its mammalian counterpart, the effect of the mouse orthologue was also analysed. Injection of murine *Grem1* RNA also caused a substantial expansion in *twist1* expression (Fig. 1H), indicating that the proteins are conserved with respect to neural crest specification. Murine *Grem1*, which escapes inactivation by the *Xenopus*-specific morpholino due to mismatches in the sequence flanking the start codon, was subsequently used for rescue experiments. Compared to embryos injected with a control morpholino (Fig. 1I), bilateral knockdown caused a reduction in neural crest on both sides (Fig. 1J). Subsequent unilateral supplementation with murine *Grem1* successfully rescued the neural crest on the injected side (Fig. 1K). Minimal lateral shift suggested that the neural plate was not dramatically expanded, as might be expected of a generic BMP antagonist, but that Grem1 specifically affects the neural crest. The ability to rescue the morphant phenotype also demonstrated specificity of the morpholino, eliminating the possibility of off-target effects.

To ensure that this was a general effect on the neural crest, several additional markers were examined. The neural crest specifier *sox10* (Aoki et al., 2003; Fig. 1L) was also reduced after knockdown (Fig. 1M), confirming that Grem1 is required, and was correspondingly expanded by overexpression (Fig. 1N). Another general neural crest marker, *foxd3* (Sasai et al., 2001; Fig. 1O), was similarly reduced after knockdown (Fig. 1P), and was also expanded by overexpression (Fig. 1Q). This validated the importance of Grem1 for neural crest specification.

### 3.3 Grem1 is required for neural plate border formation

**Fig. 2:**
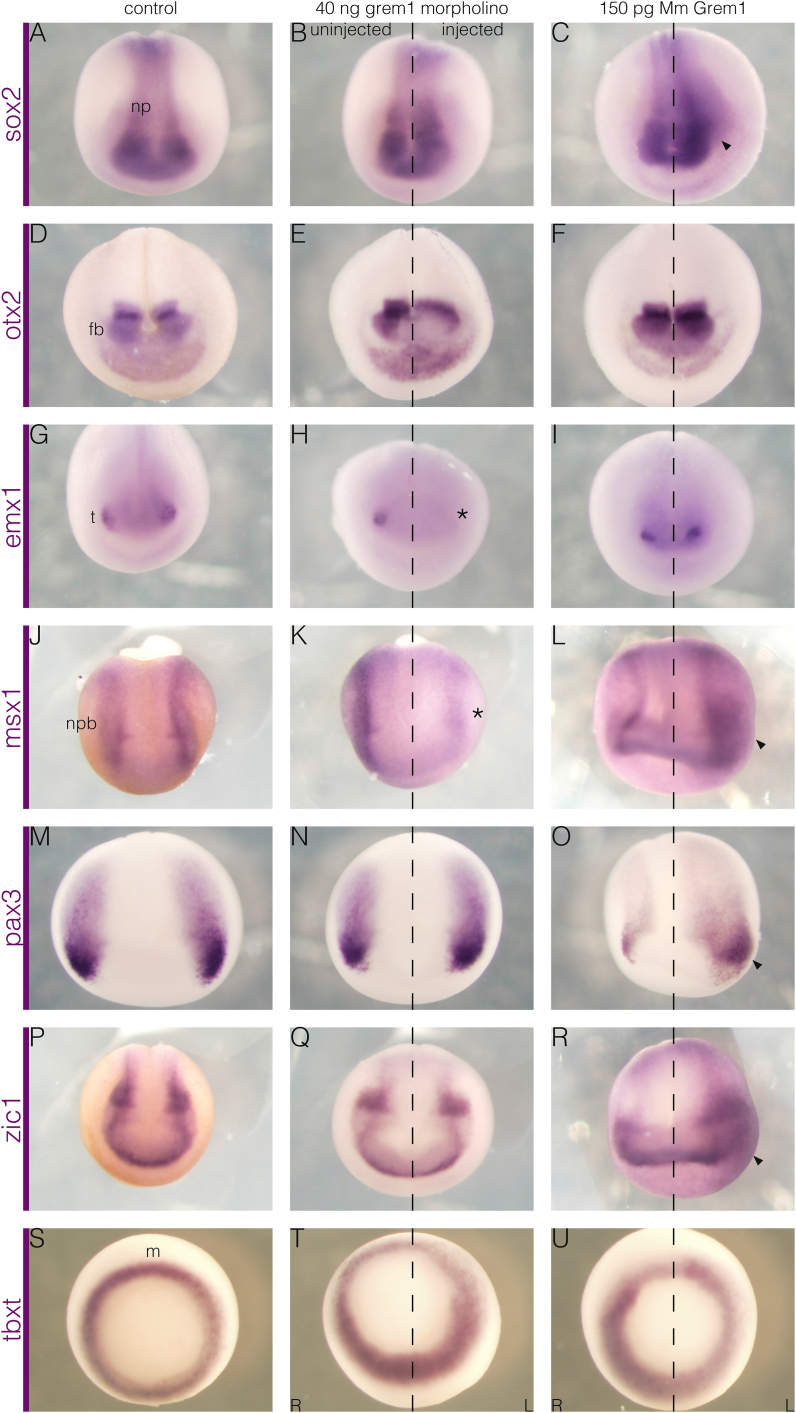
**Grem1 affects regionalisation of the ectoderm and is required for neural plate border formation.** (A, D, G, J, M, P, S) Control embryos, (B, E, H, K, N, Q, T) unilateral Grem1 knockdown, (C, F, I, L, O, R, U) unilateral murine *Grem1* overexpression. (A–I) Stage 17 embryos assayed for neural plate markers, (J–R) stage 14 embryos assayed for neural plate border markers, and (S–U) stage 11 embryos assayed for mesoderm markers. (A–C) The neural plate (np) expresses *sox2*, which appears to be unaffected by control morpholino (n ​= ​25/25) or loss of Grem1 (n ​= ​26/26) but is expanded (arrowhead) by overexpression (n ​= ​20/33). (D–F) Prospective midbrain and forebrain (fb) regions express *otx2*, which is unaffected by control morpholino (n ​= ​24/24), while the most anterior part is reduced by knockdown (n ​= ​5/25). It is unaffected by Grem1 overexpression (n ​= ​16/16). (G–I) The future telencephalon (t) expresses *emx1*, which is unaffected by control morpholino (n ​= ​17/17), requires Grem1 (asterisk, n ​= ​17/25), but is unaffected by overexpression (n ​= ​18/18). (J–L) The neural plate border (npb) is marked by *msx1*, which is unaffected by control morpholino (n ​= ​19/19), lost (asterisk) after Grem1 knockdown (n ​= ​30/45), and expanded (arrowhead) by overexpression (n ​= ​21/26). (M–O) It is also marked by *pax3*, which is unaffected by control morpholino (n ​= ​19/19), does not require Grem1 expression (n ​= ​21/23), but can be similarly expanded (arrowhead) by overexpression (n ​= ​20/24). (P–R) Likewise, *zic1*, which is expressed additionally in the anterior neural fold, is unaffected by control morpholino (n ​= ​18/18) or knockdown (n ​= ​25/25) but expanded (arrowhead) by exogenous Grem1 (n ​= ​21/23). (S–U) The mesoderm (m) expresses *tbxt* (S), which is unaffected by either Grem1 knockdown (n ​= ​8/8) or overexpression (n ​= ​8/8). Embryos in A-R are shown in frontal view with dorsal-posterior towards the top. Embryos in S–U are shown in vegetal view.

Neural crest induction is part of the more general process of ectoderm regionalisation. The effect of Grem1 knockdown and overexpression on the neural crest prompted an analysis of its effects on other genes involved in ectoderm patterning. The neural plate expresses *sox2* (Mizuseki et al., 1998; Fig. 2A), and, unlike *twist1*, *sox10*, and *foxd3*, was maintained after knockdown (Fig. 2B). Although *sox2* was expanded by overexpression (Fig. 2C), the expansion was less pronounced than those of the neural crest markers. Grem1 is therefore sufficient but not required for neural induction.

In addition to this general marker, some more specific genes were examined to assess any changes along the anteroposterior axis. Expression of *otx2* in the forebrain and midbrain (Pannese et al., 1995; Fig. 2D) was moderately reduced in the most rostral part after Grem1 knockdown (Fig. 2E), but overexpression had no clear effect (Fig. 2F). Expression of *emx1* in the anterior forebrain (Pannese et al., 1998; Fig. 2G) was lost after knockdown (Fig. 2H), but also unaffected by overexpression (Fig. 2I). These results suggested that Grem1 is also required for the specification of the anterior neural plate. The effect on both the neural crest at the lateral border and the anterior forebrain at the rostral border may reflect a general requirement for neural plate border specification. The ability of exogenous Grem1 to affect *sox2* (Fig. 2C) but not *otx2* (Fig. 2F) or *emx1* (Fig. 2I) indicates that the expanded neural plate possesses altered positional identity.

To determine the position of Grem1 in the neural crest induction cascade, several upstream markers of the neural plate border were examined. Expression of *msx1* in the neural plate border (Suzuki et al., 1997; Fig. 2J) was reduced after knockdown (Fig. 2K) and expanded after overexpression (Fig. 2L). Expression of *pax3* in the anterior neural plate border (Bang et al., 1997; Fig. 2M) was unaffected by loss of Grem1 (Fig. 2N) but also expanded by overexpression (Fig. 2O). Likewise, *zic1*, which is additionally expressed in the anterior neural fold (Mizuseki et al., 1998; Fig. 2P), was unaffected by knockdown (Fig. 2Q) but expanded by exogenous Grem1 (Fig. 2R). The expansion of all these markers after overexpression demonstrates that Grem1 is sufficient to expand the neural plate border. The effect of knockdown on *msx1* (Fig. 2K), *foxd3* (Fig. 1P) *sox10* (Fig. 1M), and *twist1* (Fig. 1G,J), indicates a fundamental requirement in the neural crest induction programme at this region.

### 3.4 Grem1 is not required for mesoderm specification

The mesoderm marker *tbxt* (also known as *brachyury* or *xbra*, Smith et al., 1991; Fig. 2S) was also examined to determine whether the effect of Grem1 on the neural crest and neural plate border was due to defects in mesoderm specification impairing signalling to the ectoderm. No clear change was seen after either Grem1 knockdown (Fig. 2T) or overexpression (Fig, 2U). This implies that Grem1 is not required for mesoderm specification.

### 3.5 The activity of Grem1 *in vivo* is dependent on HSPG binding

**Fig. 3:**
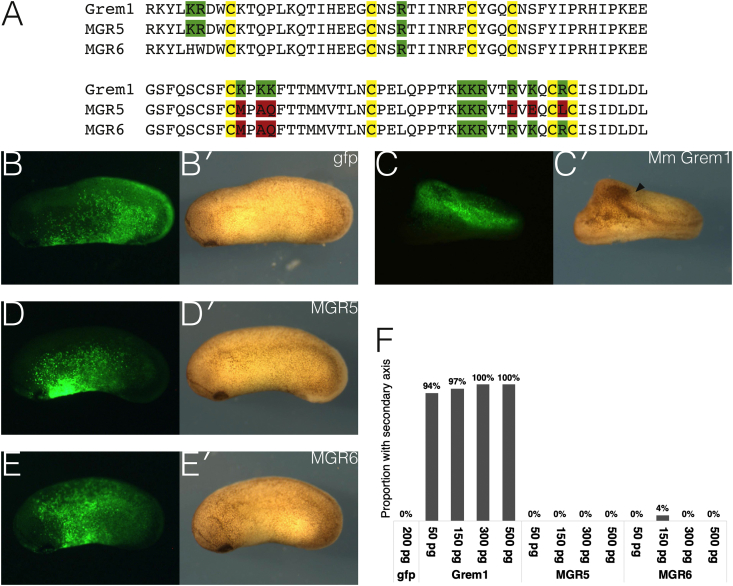
**The activity of Grem1 *in vivo* is dependent on HSPG binding.** (A) Sequence alignment of the cysteine knot domains of the wild-type mouse protein and two mutant Grem1 (MGR) constructs with impaired HS binding. Residues of the characteristic eight-membered cysteine knot are highlighted in yellow, with key basic residues mediating HS affinity in green, and non-conservative substitutions of these residues in red. (B–F) Axis induction assay by ventral misexpression of the indicated genes, traced by coinjected *gfp* (B–E), and assessed morphologically (B′-E’) for formation of a conjoined secondary axis. (B) Injection of *gfp* alone has no effect. (C) 150 ​pg *Grem1*, as expected, is able to induce a secondary axis (arrowhead). (D) This ability is abolished in *MGR5*. (E) The same goes for *MGR6*. (F) Graph summarising results of axis induction assay across a range of doses: 200 ​pg *gfp* (n ​= ​0/92), 50 ​pg *Grem1* (n ​= ​73/78), 150 ​pg *Grem1* (n ​= ​31/32), 300 ​pg *Grem1* (n ​= ​7/7), 500 ​pg *grem1* (n ​= ​3/3), 50 ​pg *MGR5* (n ​= ​0 /41), 150 ​pg *MGR5* (n ​= ​0/55), 300 ​pg *MGR5* (n ​= ​0/24), 500 ​pg *MGR5* (n ​= ​0/18), 50 ​pg *MGR6* (n ​= ​0/39), 150 ​pg *MGR6* (n ​= ​2/49), 300 ​pg *MGR6* (n ​= ​0/27), 500 ​pg *MGR6* (n ​= ​0/31). All embryos are oriented with anterior towards the left.

Grem1, like many secreted signalling proteins, is able to bind cell surface HSPGs, which can modulate its biological activity (Chiodelli et al., 2011; Tatsinkam et al., 2015, 2017). Two mutant GREM1 (MGR) proteins with impaired HS binding have been well-characterised. Their expression levels in cell culture and their affinity to BMP4 were comparable to wild-type GREM1 (Tatsinkam et al., 2015, 2017). These were used here to investigate the impact of HSPGs on the activity of Grem1 *in vivo*. The mutants contain non-conservative substitution mutations in key arginine and lysine residues, within the mapped clusters that mediate HS binding (Fig. 3A).

*Xenopus laevis* has proven to be an invaluable model organism for linking molecular function to biological relevance. Indeed, Grem1 was originally identified and defined as a BMP antagonist by an axis induction screen in *Xenopus* (Hsu et al., 1998). This approach was used again here to compare the mutants with the wild-type protein. Embryos were injected with RNA into a ventral blastomere at the four-cell stage and assessed at tailbud stages for the presence of a secondary axis. Coinjected *gfp* was used as a lineage tracer and alone had no effect (Fig. 3B,B′). Wild-type GREM1, as expected, was able to induce a secondary axis (Fig. 3C,C′), a readout for BMP antagonism. However, neither MGR5 (Fig. 3D,D′) nor MGR6 (Fig. 3E,E′) could induce a secondary axis at any of the tested doses (Fig. 3F). Independently from its affinity to BMPs, therefore, the ability of Grem1 to efficiently inhibit BMP signalling relies on unimpaired HS binding. HSPGs may facilitate its activity directly at the cell surface or alter its distribution and availability in the extracellular space. Experiments in cell culture have shown a modest reduction in BMP inhibition by MGR5 and MGR6 compared with wild-type GREM1, however they have both conserved their BMP activity and still function as BMP antagonists (Tatsinkam et al., 2017). The present findings indicate that HSPGs are essential for the neural crest inducing activity of Grem1 *in vivo*.

### 3.6 HSPG binding is required for neural crest but not neural plate border specification by Grem1

**Fig. 4:**
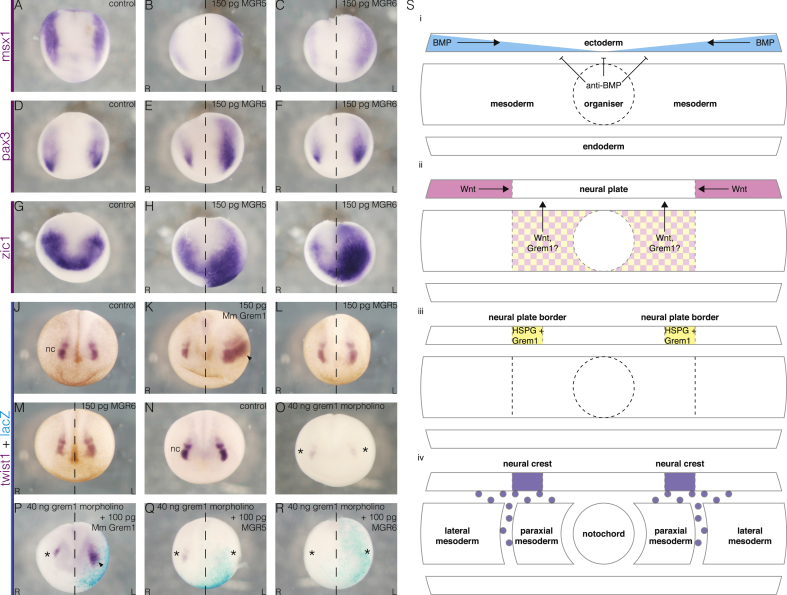
**HSPG binding is required for neural crest but not neural plate border specification by Grem1.** (A–I) Stage 14 embryos assayed for neural plate border markers. (A, D, G) Control embryos, (B, E, H) unilateral *MGR5* expression, (C, F, I) unilateral *MGR6* expression. (A–C) *msx1* expression at the neural plate border is unexpectedly expanded and upregulated by both *MGR5* (n ​= ​6/8) and *MGR6* (n ​= ​7/9). (D–F) *pax3* is similarly affected by *MGR5* (n ​= ​7/9) and *MGR6* (n ​= ​5/9). (G–I) The same goes for *zic1*, with *MGR5* (n ​= ​7/7) and *MGR6* (n 7/7). (J–R) Stage 18 embryos assayed for *twist1* expression. (J) The cranial neural crest (nc) is marked by *twist1*. (K) As expected, unilateral overexpression of *grem1* RNA, expands the neural crest on the injected side (arrowhead). (L–M) The same dose of *MGR5* (L, n ​= ​26/26) or *MGR6* (M, n ​= ​29/29) is unable to elicit an expansion. (N–R) Neural crest induction rescue experiment. (O) Bilateral Grem1 knockdown severely impairs neural crest formation (asterisks). (P) As before, unilateral supplementation with 100 ​pg wild-type mouse *grem1* restores neural crest development (arrowhead). (Q–R) Accordingly, the same dose of *MGR5* (Q, n ​= ​17/17) or *MGR6* (R, n ​= ​15/16) is unable to rescue the neural crest. (S) Schematic summarising a working model for the function of Grem1 in neural crest induction. (i) BMP (blue) antagonism from the organiser initially induces neural identity in the ectoderm. (ii) Wnt (pink) from the underlying mesoderm and non-neural ectoderm, and Grem1 (yellow), perhaps also from the underlying mesoderm, signals to the future neural plate border, independently of HSPGs. (iii) HSPGs in the neural plate border stabilise and/or modulate the activity of Grem1 protein in this tissue. (iv) This enables the eventual induction of the neural crest. Embryos are shown in frontal view with dorsal-posterior towards the top.

The requirement for Grem1 to bind HSPGs to induce neural plate border was also investigated. As in the earlier experiments (Fig. 2J-R), embryos were analysed at stage 14 for *msx1* (Fig. 4A), *pax3* (Fig. 4D), and *zic1* (Fig. 4G) expression. Unexpectedly, in contrast to their loss of function in the axis induction assay (Fig. 3), both constructs retained the ability to expand the expression of *msx1* (Fig. 4B-C), *pax3* (Fig. 4E-F), and *zic1* (Fig. 4H-I). Effective HSPG binding, therefore, is not required for the initial inductive capacity of Grem1 at the neural plate border.

The effect of the mutants on the neural crest itself was then tested. As before (Fig. 1D–K), embryos were analysed at the neurula stage for *twist1* expression (Fig. 4J). Again, unilateral Grem1 overexpression caused an expansion on the injected side (Fig. 4K). However, neither MGR5 (Fig. 4L) nor MGR6 (Fig. 4M) could do the same. Similar results were found for *sox10* (Figs. S2A–C). However, *foxd3* was affected by MGR5 and MGR6 in a minority of cases (Figs. S2D–F). This may represent a more upstream position in the neural plate border and neural crest induction cascade. These findings were supported by attempts to rescue neural crest induction (Fig. 4N–R). As in the previous rescue experiment (Fig. 1I–K), bilateral morpholino knockdown caused a severe reduction in expression (Fig. 4O), but subsequent unilateral injection of murine *Grem1* was sufficient to restore it again (Fig. 4P). Again, neither MGR5 (Fig. 4Q) nor MGR6 (Fig. 4R) could do the same. This demonstrated that functional HSPG binding is essential for the capacity of Grem1 to induce neural crest, for which it is endogenously required.

The effect of the mutants on the neural plate border was confirmed by a similar rescue experiment (Figs. S2G–K). Bilateral knockdown obliterated *msx1* expression (Fig. S2H), which was partially rescued by *Grem1*, as expected (Fig. S2I). Interestingly, both MGR5 (Fig. S2J) and MGR6 (Fig. S2K) were also able to rescue *msx1*. This confirmed that HSPG binding is not required for the upstream activity of Grem1 at the neural plate border.

## 4 Conclusion

The experiments presented in this study demonstrate a novel role for Grem1 in neural crest induction. Furthermore, they show that this function is regulated by HSPGs. Grem1 acts independently of HSPGs to first induce neural plate border genes such as *msx1*. It then enters a second, HSPG-dependent phase to allow the expression of neural crest specifiers (Fig. 4S). The results of this study highlight the importance of interactions between HSPGs and components of key signalling pathways during embryonic development.

Whether HSPGs simply stabilise Grem1 protein sufficiently to maintain its inductive activity, or whether they also modulate its interactions with other molecules remains to be seen. For example, HSPGs have been shown to mediate Vegfr2 agonism by Grem1, indicating that they can impact its effects on pathways other than BMP signalling (Chiodelli et al., 2011). Furthermore, VEGF signalling has previously been linked to neural crest migration so it is reasonable to suggest that HSPG-dependent VEGF signalling may be involved in other aspects of neural crest development, such as induction (McLennan et al., 2015). Our data suggest that the effect of Grem1 on BMP signalling and potentially VEGF and other pathways is dependent on the presence and perhaps the composition of surrounding HSPG molecules. This study represents the first evidence of a role for Grem1 in neural crest specification and reveals a novel role for HSPGs during this process.

## Funding sources

This research was funded by the 10.13039/501100000265Medical Research Council (G0901899).
