# Supplementary material for: Heparan sulfate proteoglycans regulate BMP signalling during neural crest induction
Source: Dev Biol. 2020 Apr 15;460(2):108–14. doi: 10.1016/j.ydbio.2019.12.015 (PMC7196931; doi:10.1016/j.ydbio.2019.12.015)
Supplement: Multimedia component 2 [file mmc2.docx]

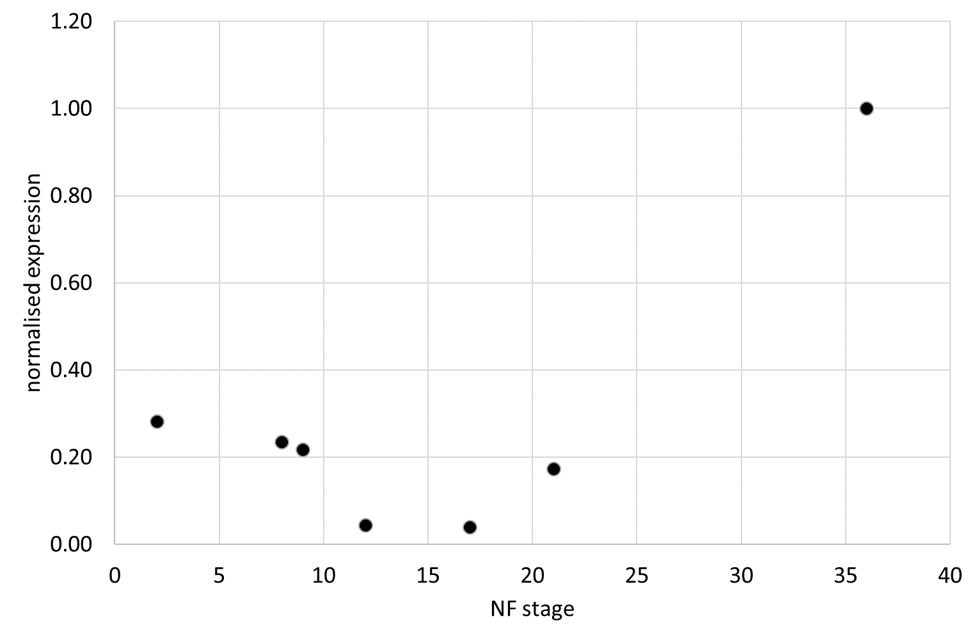


**Figure S1. Temporal expression profile of Grem1.** qPCR of whole embryos shows an early peak of expression before gastrulation, a slight decrease, then a gradual increase into tailbud stages. NF stage, Nieuwkoop and Faber stage.

**Figure S2. Dependence of Grem1 HSPG binding on neural crest and neural plate border specification.** (A-F) stage 17 embryos assayed for neural crest markers. (A-C) *sox10* expression in the neural crest (nc) is unaffected by MGR5 (n = 8/8) and MGR6 (n = 9/9). (D-F) *foxd3* is variably expanded (arrowheads) by MGR5 (n = 4/20) and MGR6 (n = 5/20). (G-K) stage 15 embryos assayed for neural plate border (npb) marker *msx1*. (H) Bilateral Grem1 knockdown severely reduces expression (asterisks). (I) Unilateral injection of wild-type mouse *grem1* partially restores (arrowhead) expression levels (n = 5/7). (J-K) This ability is retained by Mgr5 (n = 2/3) and Mgr6 (n = 7/7). Embryos are shown in frontal view with dorsal-posterior towards the top.
